# How young people perceive change to occur in family therapy for anorexia nervosa: a qualitative study

**DOI:** 10.1186/s40337-024-00971-8

**Published:** 2024-01-22

**Authors:** Julian Baudinet, Ivan Eisler, Anna Konstantellou, Mima Simic, Ulrike Schmidt

**Affiliations:** 1 Centre for Research in Eating and Weight Disorders, Institute of Psychiatry, Psychology and Neuroscience, King’s College London, De Crespigny Park, London, SE5 8AZ UK; 2 Maudsley Centre for Child and Adolescent Eating Disorders, South London and Maudsley NHS Foundation Trust, De Crespigny Park, Denmark Hill, London, SE5 8AZ UK; 3 Adult Eating Disorders Service, South London and Maudsley NHS Foundation Trust, Denmark Hill, London, SE5 8AZ UK

**Keywords:** Anorexia nervosa, Adolescents, Family therapy, Family based treatment, Maudsley family therapy, Qualitative

## Abstract

**Background:**

Family therapy for anorexia nervosa (FT-AN) is the first line recommended treatment for child and adolescent anorexia nervosa. Despite evidence of its efficacy, little is understood about the treatment mechanisms. This study aimed to understand how young people who have received FT-AN perceive change to occur across treatment.

**Method:**

Fifteen adolescents (age 12–18 years) completed individual semi-structured interviews online. Recordings were transcribed verbatim and analysed using reflexive thematic analysis.

**Results:**

Four inter-connected themes describing the process of change during treatment were generated; (1) relationships as the vehicle for change, (2) an awakening, (3) through, not around – no way out, (4) the life beyond.

**Conclusions:**

Current data match relatively closely with theoretical models of FT-AN and emphasise the importance of building trust with all family members, including the young person. Additionally, supporting the family to create a trusting context in which there is a sense that the only way out of the illness is by going through it (rather than avoiding it) is critical. Empirical investigation of each of the described mechanisms is needed.

## Abstract (plain language summary)

Family therapy for anorexia nervosa (FT-AN) is the first line recommended treatment for child and adolescent anorexia nervosa. While outcomes are generally good, little is understood about how the treatment works. This study aimed to understand how young people who have received FT-AN perceive change to occur across treatment. Fifteen adolescents (age 12–18 years) completed individual interviews online. All interviews followed a similar structure, with recordings transcribed word-for-word and analysed using a methodology called reflexive thematic analysis. Four inter-connected themes were generated from the interviews; (1) relationships as the vehicle for change, (2) an awakening, (3) through, not around – no way out, (4) the life beyond. These themes match relatively closely with descriptions of FT-AN theory and emphasise the importance of building trust with all family members in treatment, including the young person. Additionally, supporting the family to create a trusting context in which there is a sense that the only way out of the illness is by going through it (rather than avoiding it). More data are needed to test these ideas empirically and with other populations.

## Background

Eating disorder focussed family therapy is the current first line recommended treatment internationally for adolescent anorexia nervosa (Hilbert et al. 2017; NICE 2017). Several different versions of eating disorder focused family therapy exist and go by various names, including Maudsley family therapy for anorexia nervosa (FT-AN; Eisler et al. 2016), family-based treatment (FBT; Lock and Le Grange 2012) and parent-focussed treatment (PFT; Le Grange et al. 2016). While these treatments are more similar than different, they vary somewhat in the number of phases described, their emphasis on engagement, the use of formulation, the raising of parental anxiety and the inclusion of individual sessions with the young person within the models (Gorrell et al. 2023). While outcomes at end of treatment (Jewell et al. 2016; Lock and Le Grange 2019; Simic et al. 2022) and follow-up (Eisler et al. 2007; Stewart et al. 2022) are encouraging, a significant minority of young people require more or alternative treatment.

To date, relatively little attention has been given to the experience of treatment from the young person’s perspective or their perception of how change occurs during treatment. Qualitative data collected on the experience of treatment suggest it is perceived as helpful, supportive, lifesaving, yet also very distressing for both young people and parents/caregivers (cf. Medway and Rhodes 2016 for a narrative review). Despite this reported distress, it has also been associated with an increased closeness within familial relationships across the treatment journey. In a qualitative study with 16 adolescents and 28 of their parents who had a ‘good’ outcome in FBT, Wallis et al. (2017) found that prior to treatment families were experiencing distress, disconnection and significant conflict. Across treatment, via the FBT treatment structure, therapist support and medical setting, a process of ‘relational containment’ was created. Parents could trust the therapist and treating team, which helped them to feel more confident and hopeful in the recovery process. This, in turn, supported hope and recovery-oriented change for the young person (Wallis et al. 2017).

These data suggest early containment of anxiety, trust within the parenting team and parent-child relationships, and the generation of hope are important ingredients to a good outcome. This fits with quantitative findings demonstrating that increased conflict and expressed emotion may be associated with a poorer treatment response (Gorrell et al. 2022) and that parents who report fewer positive caregiving experiences may be better suited to multi-family, rather than single-family, therapy in which greater intensity and support can be offered (Baudinet et al. 2023b).

Data from young people and families for whom treatment has not been sufficient are also important to consider. FBT in this context has been described as overly behavioural and lacking flexibility (Medway and Rhodes 2016). It has also been suggested that focusing on the therapeutic alliance and tailoring treatment to the individual is valued and considered important (Williams et al. 2020). Conti et al. (2017, 2021) completed interviews with people who had either dropped out of FBT or continued to be distressed post-discharge. They found that, while young people reported the treatment to be lifesaving and family support helpful, many experienced a loss of voice and identity during treatment, especially within the early stages that require parents to take a lead. It was felt that being overly parent focused and delaying offering skills to the young person to manage distress contributed towards this loss and exacerbated distress (Conti et al. 2021).

These data speak to the importance of engaging young people as well as parents from the very beginning of treatment, which has not been a priority in theoretical descriptions of FBT. In a recent conceptual description of FBT and a comparison to Enhanced Cognitive Behavioural Therapy (CBT-E), it was noted that adolescents are ‘not actively involved’ in FBT (Dalle Grave et al. 2019).

The importance of engagement and the therapeutic alliance has been described and empirically evaluated in the broader eating disorder literature. Systematic reviews and meta-analyses indicate that engagement and therapeutic alliance are associated with improved outcome (Graves et al. 2017; Werz et al. 2022) and that early change is predictive of end-of-treatment response (Chang et al. 2021; Gorrell et al. 2022; Hamadi and Holliday 2020; Linardon et al. 2016; Vall and Wade 2015). In one meta-analysis, it was found that the therapeutic alliance was particularly important for younger people, for whom alliance had a stronger relationship to outcome than for older people (Graves et al. 2017). Within FBT/FT-AN studies specifically, one study found that parent alliance with the therapist is strong, but did not predict end of treatment outcomes (Forsberg et al. 2014), whereas others have found that early alliance is associated with improved outcomes (Jewell et al. 2021; Pereira et al. 2006) and reduced dropout rates (Pereira et al. 2006). Nevertheless, most of these data are correlational and given the likely bidirectional process that exists between alliance and outcome, it is hard to determine causal associations (Cuijpers et al. 2019).

In response to these findings, Maudsley FT-AN has been updated to specifically emphasise the engagement of all family members from the outset of treatment, including the young person, and the use of formulation to ensure treatment is individually tailored (Baudinet et al., 2021a, b). What remains unclear is what impact this has on treatment experience and outcomes, and whether young people perceive this to impact or promote recovery-oriented change.

The study aims to explore the perceived change mechanisms of FT-AN from the young person’s perspective. This will help shed light on possible change processes and treatment mechanisms within FT-AN and identify future areas for empirical investigation.

## Methods

### Sample

Young people (12–18 years) were eligible for this study if they (a) had a diagnosis of anorexia nervosa or atypical anorexia nervosa, (b) received FT-AN as part of their outpatient treatment at the Maudsley Centre for Child and Adolescent Eating Disorders (MCCAED) during the recruitment period. MCCAED is a large, specialist child and adolescent eating disorder services in London, UK. The service has a catchment area of over two million people and sees people up to the age of 18 years. Potential participants were identified at the point of discharge from treatment by the clinical team, who initially told potential participants about the research study. Once the research group received consent to contact, up to three attempts at contact were made per person.

### Recruitment

All young people who completed FT-AN at MCCAED between July 2022 and February 2023 were approached to participate, regardless of treatment outcome, length or engagement. Qualitative interviews were conducted with all participants individually for those who provided consent (> 16 years) or assent (< 16 years). All interviews were conducted via video-call by authors JB (male, cisgendered, white Australian, clinical psychologist, DClinPsych, extensive experience of FT-AN) and AK (female, cisgendered, assistant psychologist, PhD, extensive theoretical knowledge, but not clinical experience, of FT-AN). Each interview lasted approximately 60 m with only the interviewer and the participant present. Interviews were recorded and then transcribed verbatim. All interviews followed the same topic guide. Transcriptions were not returned to participants for review.

For participants where JB had been involved in treatment delivery, interviews were completed by AK. To minimise bias, all transcripts were de-identified during the transcription process conducted by a transcription service, meaning deidentified transcripts were used during the analysis process. Participants were informed at the beginning of their interview that the aim of the research was to explore their experience of how young people perceive change to occur during treatment.

### Diagnostic assessment and screening

All young people completed the Development and Well-being Assessment (DAWBA) (Goodman et al. 2000) prior to attending their initial assessment with the service. The DAWBA is a structured diagnostic tool that generates DSM-5 (American Psychiatric Association, 2013) and ICD-10 (World Health Organization 2004) predicted psychiatric diagnoses for two to seventeen-year olds. Eating disorder diagnosis was confirmed at the MCCAED clinical assessment.

### Treatment description

All participants in this study received manualised outpatient FT-AN (Eisler et al. 2016) as part of routine clinical care in a public outpatient clinic. FT-AN is a phased treatment, which begins with a focus on engagement (phase 1) and symptom management (phase 2), followed by support with broader adolescent and family lifecycle difficulties (phase 3) and therapeutic work around ending and relapse prevention (phase 4). Early in treatment, sessions are offered weekly, which moves to fortnightly and beyond as treatment progresses. The early phases of treatment have a strong focus on practically supporting young people and families to manage the eating disorder symptoms, gain weight (if required) and build skills around tolerating related distress. The content of sessions in the later phases is individualised to each family. Common themes include returning to independent eating, managing school and peer relationships, tolerating uncertainty, etc. The number and frequency of FT-AN sessions are not pre-determined, rather they are based on family need and clinical presentation.

### Analysis plan

Data generated were analysed by authors JB and AK using reflexive thematic analysis (Braun and Clarke 2020). This was conducted within a critical realist framework, from which experience and meaning are considered subjective and influenced by social and cultural context. The analysing authors approached the data with extensive knowledge of FT-AN, although AK had no clinical experience delivering the treatment.

The analysing authors followed the six phases of reflexive thematic analysis (Braun and Clarke 2006). This included an initial period of immersion in the data followed by each independently generating codes and preliminary themes. Via an iterative process over two separate one-hour meetings, themes were then generated and revised to reach a final consensus. There was no difficulty in reaching consensus with initial codes and themes that were generated individually easily mapping onto and complimenting each other.

A deductive and flexible approach (Fletcher 2017) was used during analysis that drew on existing FT-AN and systemic theory and literature. Given the authors’ knowledge of the treatment, some concepts were expected to emerge, e.g. the need for firm parental support early in treatment. While this inextricably influenced the analysing authors’ approach to the data, coding was not constrained by these expectations. The concept of data saturation was not used during the analysis, as it was recognised that different meanings are generated by different researchers and the process is inescapably subjective (Braun and Clarke 2021). No software was used to assist analysis.

### Member checking

Member checking was completed in line with current guidance to add rigour to the findings (Birt et al. 2016). All young people were provided with a summary of the results and asked two questions; *(1) Do the themes and findings fit your experience and understanding of how change occurred during your treatment?, (2) Would you add/amend/change/delete anything? Please elaborate.* After a maximum of three attempts at contact, three young people (3/15, 20.00%) responded with feedback via email. All three young people who responded said the results fitted their experience well. One suggested emphasising the importance of psychoeducation about weight gain preceding broader symptom recovery.

## Results

A total of 46 potentially eligible young people were approached to participate. Of these, 15/46 (32.61%) consented and participated, 12/46 (26.09%) actively declined, 14/46 (30.43%) passively declined (provided initial consent or requested further information but were unreachable or unavailable to complete an interview thereafter), and 5/46 (10.87%) could not be reached with a maximum of three attempts at contact. Reasons for non-participation included not being interested in the study and not wanting to revisit the illness/treatment journey.

### Participants

Most participants identified as cisgendered female (13/15, 93.3%) and White (White British = 9/15, 60.00%; White Other = 2/15, 13.33%; Black British = 1/15, 6.67%; Hispanic = 1/15, 6.67%; Mixed = 1/15, 6.67%; prefer not to say = 1/15, 6.67%).

One (1/15, 6.67%) participant identified as male and one (1/15, 6.67%) as gender fluid. One (1/15, 6.67%) identified as neurodiverse. None identified as living with a disability. Mean age at interview was 16.23 years (sd = 1.48, range = 13–19). All (15/15, 100%) were living at home at the time of study participation. Ten (66.67%) were living with two parents in the one home, and five (33.33%) had parents who were separated.

At initial assessment with MCCAED, mean age was 15.24 years (sd = 1.32, range = 13–18). The majority of young people (9/15, 60.0%) met DSM-5 criteria for anorexia nervosa (restrictive subtype = 8, binge-purge subtype = 1), and six (6/15, 40.0%) for Other Specified Feeding or Eating Disorder (OSFED, atypical anorexia nervosa). Mean percentage median body mass index (%mBMI) was 86.60% (sd = 9.98, range = 70.00–105.39) at assessment and 94.39% (sd = 6.96, range = 81.00–105.66) at discharge. Nine (60.0%) of the participants had two or more predicted DSM-5 comorbid diagnosis on the DAWBA (zero comorbidities = 1, 1 comorbidity = 0, 2 comorbidities = 4, 3 comorbidities = 3, 4 + comorbidities = 2, missing = 5).

The participating young people had a relatively heterogeneous treatment course. The mean duration of treatment was 7.21 months (sd = 3.93; median = 6.28), however it ranged from 3 to 18 months. Mean number of sessions attended was 15 (sd = 6.85; median = 14, range = 5–30).

Young people from participating (*n* = 15) and eligible non-participating families (*n* = 31) did not significantly differ in age (mean_participants_ = 15.24 [sd = 1.32], mean_non-participants_ = 15.21 [sd = 1.75] years, *t*(44) = 1.27, *p* = .90), treatment duration (mean_participants_ = 7.21 [sd = 3.93], mean_non-participants_ = 8.89 [sd = 3.62] months, *t*(44) = 1.44, *p* = .16), weight at initial assessment (mean_participants_ = 86.60 [sd = 9.98], mean_non-participants_ = 83.18 [sd = 8.12] %mBMI, *t*(44) = 1.24, *p* = .22) or discharge (mean_participants_ = 94.39 [sd = 6.96], mean_non-participants_ = 95.06 [sd = 6.76] %mBMI, *t*(42) = 0.30, *p* = .76), tested using independent samples t-tests.

### Qualitative findings

**Fig. 1:**
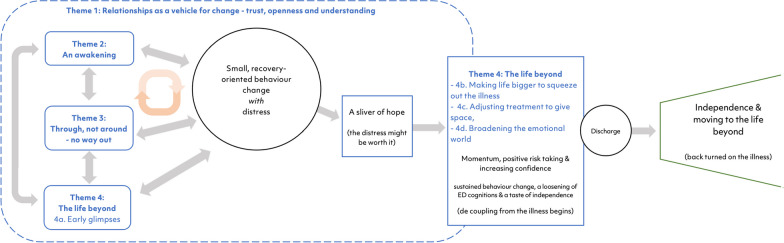
Possible model of change processes during FT-AN from the young person perspective

Four themes were generated and are understood to be inter-connected. The way these combined to generate a possible model of change are presented in Fig. 1. This is a speculative model for future investigation that is inherently influenced by the analysing authors and their own experiences and biases.

The first theme (Theme 1: relationships as a vehicle for change) was described as creating a foundation, or a safe base, from which the other themes were enacted. The next three themes (Theme 2: an awakening; Theme 3: through, not around – no way out; Theme 4: The life beyond) combined to promote small recovery-oriented changes through repetition and perseverance. Once the young people perceived recovery as the only way forward, hope and acceptance were described as increasing. This led to momentum, increased confidence and the ability to begin the process of developmentally appropriate independence and leave the illness behind. It was in these latter parts of treatment that theme 4 (the life beyond) came more into the forefront, during which young people began to take more positive risks and families learnt to trust each other again.

I think early treatment was very much nutrition focused. It was kind of let’s just get her fed enough. And then middle treatment was kind of unpacking lots of fears. And I don’t know, just things like thoughts around food, which had already kind of died down a little bit by that point. And then end of treatment for me was kind of looking at what might have caused it, how you can prevent it. But I think what really helped as well was kind of learning about the process as you go along … that was really helpful for me.

### Theme 1: relationships as the vehicle for change

Relationships were described as strongly influencing, and being influenced by, the treatment and recovery process. Relationships with parents and therapists were most discussed, however other family members, friends, partners and other professionals (e.g. dietitians, teachers) were also described as important. Almost all young people said they would not have made the same progress, nor would it have been as quick, without their parents involved in their treatment. As one young person put it: “*I don’t think I would have been able to do it by myself.”*

There was a sense that having people who are close to you trying to support helped the young person to push past moments when they were facing illness-related cognitions that felt too overwhelming or too difficult to manage alone.

I just don’t think it’s possible to recover without a support system around you, without people, helping you pick foods, helping you sit down at dinner and be like: ‘okay, you gotta eat this’ … they’re the ones that are fighting the eating disorder’s voice in you. And there’s a point where the eating disorder’s voice is stronger than anything else in you. And so you are not physically able to fight that yourself. You need help. You need other people to, like, to help fight that voice until you’re capable of doing that yourself.

The young people described how just having parents or supports attempt to help and continue to show support, in itself, was a powerful message of care. This was the case even when the support was not always easy to accept. Most described how difficult it was to directly ask for help and spoke about often actively rejecting it. One young person said: “*it’s really hard in that moment to try and tell people that you want to get better.”* Having parents learning to take a calm and kind approach, attempting to understand (even if they were struggling to) and consistently ‘showing up’ despite difficulties, were specifically described as helpful.

If you were, like, having a really hard time and then, like, the person who’s meant to care about you, like, the literal most in the entire world was just like, not, like, caring or like, just couldn’t be bothered to even try and help. That would really be a hindrance to treatment or recovery to be honest.

There was also a sense that involving families in treatment could be as important for them as it was for the young person themselves. Essentially, it was the relationships that acted as the vehicle through which progress could be enhanced or hindered. Through parental attendance and support from the multi-disciplinary team (MDT), parents were able to understand more and approach the situation more effectively.

*“And so it’s like as much as it was about teaching me the correct thoughts, and the correct patterns, and about how much it was teaching me how to get better. It was also teaching my parents what the right things were to say and what the right behaviours were, and what actually they had done that was maybe not so healthy. And so I think it’s, I think having parents involved is just as important.“*.

Relatedly, there was a sense from the young people that relationships changed during and as a result of the treatment. Several young people described feeling closer as a family at the end of treatment.

It helped to kind of explore an emotional side and helped to gain an understanding of different feelings and being able to just be open, because I think before things weren’t always discussed and talked about. It was just maybe, like a hug as comfort. But now I think we’ve gained both elements, as in comfort and the ability to actually talk about things together.

The importance of trust was also described as one of, or perhaps the most, important relational factors in promoting change. Participants said that engagement and trust had to be prioritised from the outset and described it as growing during the course of treatment. This was described as not being the same as liking the therapist or treatment. At the beginning some young people were not particularly fond of either. Being able to trust the therapist and MDT, even when they did not like them so much, was seen as the important element. As one young person put it:

I really connected with [my therapist], the person, psychiatrist. And my mum really connected with him as well, and it was funny because in the beginning of the treatment, I, you know, obviously really resented him. I was really fighting against being in treatment, you know, I was really against it, and then it was kind of like a Nanny McPhee, you know? It’s like when you need me, but don’t want me, or then it’s like, you want me, but don’t need me. By the end I loved [the therapist] so much, and I was so grateful for the experience I’ve had with him, but I didn’t need him anymore, so I kind of had to just, you know, move on.

Nevertheless, parental involvement was not always experienced as easy, and could hinder the young person’s ability to talk or be open in sessions, especially early on in treatment. This made some young people suggest more individual time with the therapist would have been helpful.

I think it was important that they [my parents] were there so that they could see kind of my struggle and also so that they could learn what was going on. They needed that information just as much as I did. But there were other times where it was just really awkward talking about my deepest, darkest anorexia secrets, I definitely was able to open up more when it was just one on one with my psychologist, about actual behaviours, because you don’t want to admit those kind of things to your parents.

Several young people spoke of early treatment sessions being very parent focused. For some, this inhibited their ability to bring some of their internal experiences to treatment and ultimately meant they struggled to engage.

I feel like a lot of the time at the beginning, I didn’t really say much at all. I feel like it was mostly my parents talking, and that was quite unhelpful.

### Theme 2: an awakening

Most young people spoke about a point in their treatment where they had a moment of clarity or insight into the fact that something was not quite right for them – that they were unwell. This was often an internal experience that was not necessarily shared with others for some time. There was also a view that this moment of insight did not always last, or persist, but was a notable turning point in the recovery journey.

I think it’s very difficult because I think a part of it has to come from yourself. And it’s very difficult to get to that point when, you know, your brain is not working and you’re not eating and it’s very hard to have that clarity. But I think, for me, it was just like [the realisation that] if I keep on this path, I’m gonna die.

How this happened, or with whom, was relatively heterogeneous. A few young people spoke about having this awakening during their initial assessment when their diagnosis was discussed, and psychoeducation was provided about the impact of the illness on their current and potential future health and functioning.

I think at the beginning to get me started and want to recover was when we had the very first session when they actually diagnose you, and then we had the doctor came and spoke to you and he was quite harsh about it and really direct, to say that if you keep going this is what could happen. And I think that just scared me a bit but I think I needed that to scare me into recovery to begin with, and then when we had the session with [therapist name] then she was really lovely and I think that I needed that throughout, I think the scare at the beginning helped but then if I had that all the way through, that wouldn’t have worked for me because I would have just been, I don’t want to do that….

One person described how their shift towards recovery came from trusting the dietitian who joined their family for consultation:

That was quite like a little turning point … when the dietitian told me that she hears all this stuff from people online who claim that this is that … but she’s [the dietitian] actually saying the actual scientific stuff, and the thing she was describing, I knew it was the truth, like solid facts. And when I was faced with a situation to eat a fear food or something, I was like, Okay, remember what the dietitian said. The dietitian said, Oh, it’s fine if you have this and this because of that and that. And, I guess, for the eating disorder, it was like the biggest thing that was able to go against these thoughts.

Another spoke about how this happened for her after seeing an old friend overseas who was also struggling with anorexia nervosa and remembered being shocked by her physical health:

When I went to [country name] she [friend] seemed so much skinnier and I was so shocked, and it kind of scared me because I knew she knew I was struggling with that, with eating. And I guess, seeing her in such a position, where I knew she didn’t have control. Like, she kind of lost control of how much she ate, and she didn’t really see an issue with it. I noticed that she wasn’t eating because I stayed over at her house and I slept there and she wasn’t eating. She wouldn’t eat, and I got scared. It was kind of the first time I actually got scared.

Others spoke of how this happened after seeing the impact and distress their family members were going through:

“There was so much conflict. And I really felt, you know, my little sister, you know, still tells me how almost traumatised she was just from seeing me, you know, me being at the table and not eating, like it was very much felt by the family. And I think I just realised that this is not the way to be.”

I remember there was a little bit of guilt for what I was putting my parents through … and in a strange way, I kind of think that guilt was a motivator as well, because I was kind of like, I cannot keep doing this to my parents. They cannot live like this forever.

While this new awareness was a specific, memorably moment for some, for others this was a more gradual process that seemed to occur through ongoing psychoeducation and increased understanding within the family. Several young people spoke about how important the realisation was that eating disorders are illnesses, rather than ‘difficult behaviour’.

They [the MDT and family] definitely saw me as a person. They definitely sort of understood how I was feeling, definitely. But it was very clear that they did show it as, I’m pretty sure it was, they saw it as a condition, you know, not a way you’re feeling. Which I think that probably helped me feel like I needed to eat because instead of it being: ’This is the way you are’, instead of it being like that. It was: “This is a thing you have, which you need to repair”. What’s the word? Recover. My [therapist] was really good at explaining to my parents and my family: ‘Actually this isn’t a choice. You know, this isn’t something [young person’s name] woke up one day and decide to do to herself, this is an illness. I’m going to treat it like an illness.’ I think having someone there to explain to my parents, someone who may have been a bit misinformed. Actually, how and why is this happening, and what we’re gonna do about it? Was so useful because I couldn’t be that person. I didn’t even really know what was going on, so that definitely was really important.

There were a small number of participants who did not describe such an awakening. For these participants, there was a sense that the illness was still valued in some way. They had managed to gain some weight and sustain their physical health independently, however, their identity seemed to still be connected with their appearance and drive for thinness.

### Theme 3: through, not around – no way out

All young people spoke about the importance of intense perseverance in the face of distress, which was usually led by parents at the beginning of treatment. With repeated, warm, firm and consistent parental support, young people spoke of coming to a feeling like there was no alternative but to recover. One young person spoke about it feeling like “*constant drilling”.* Importantly, none of the young people experienced being physically forced to eat, rather they spoke of their parents creating an environment in which eating was perceived by the young person as the only way forward.

It was very consistent … when your family is there, you sit down, you have to, you’re not allowed to leave. And it feels like it’s something you have to do … It feels like something you don’t have a choice in. Obviously, I’m glad I didn’t have a choice in it because otherwise I wouldn’t be better.

I think, personally, that it’s finding out there’s a problem, and then the person who looks after you making you eat. I think it was a bit brutal at times, going from zero to 100, three meals a day, this, that, that. I think that’s the thing that made the difference.

If the process of small steps was repeated, there was a sense from the young people that a turning point came when the illness had no escape routes; when there were no more ‘tricks’ left, and they could not avoid the task of eating and working towards recovery anymore.

I couldn’t escape, like I couldn’t go out and tell my parents I’d eaten. I wasn’t allowed to leave the house to exercise. So, like, in that sense, that really, really was very crucial for my recovery because there was no lying. It was very clear to my parents when I stopped eating because we were eating three meals a day together.

At first, understandably, this was described as very challenging, distressing and, for many, anger-inducing. It made almost every meal difficult and coming to treatment very stressful for most. One young person compared treatment to painful, life-saving surgery. Having said that, when reflecting on the treatment journey after some time had passed, this was considered a crucial step in the process of change by most and something needed in order to recover. One young person said: “*I certainly didn’t like it, but I also did need it.”.* Another said: “*It was hard, but it was necessary.”*

I think the first thing that I felt on my meal plan was like anger and also just anger at myself, because my anorexia brain was like, ‘You could have kept the secret for longer. We could have kept going’, you know what I mean. But now that I was in treatment, there was no way that I could have got out of treatment. They were not going to let me leave still this underweight. Still this fearing food. And at the same time, I was kind of like the thoughts of, You need to get your life back, started to win again. So slowly it was kind of just like, it’s painful eating this food, it’s a lot of food, but it needs to happen. Well, at first, … I was just really angry at my parents, like 24/7. I hated it. But then, later on when I made the change for myself as well as my parents, that’s when the relationship became stronger because I was talking to them about it and things like that, so.

One young person described this change mechanisms as the family needing to make recovery the top priority and only then could they begin to move to the next stage of recovery – life beyond the small steps of early-to-mid treatment.

It [recovery] became the top priority in the family … just help her recover. And then it kind of felt like [that] I think for me also.

### Theme 4: the life beyond

Young people spoke of the importance of keeping their life outside the eating disorder present in treatment. Many spoke of the way the illness made their life feel very small and narrow. People spoke of feeling very isolated and ‘shut down’. One young person said: *“for me, the anorexia kind of made me shut out a lot of my friends, and I felt like extremely lonely at the beginning of treatment”.*

#### 4a. Early glimpses

Ensuring that issues that young person faced in their everyday life not related to AN were discussed in sessions early in treatment enhanced young person’s motivation to endure distress related to eating, weight restoration and recovery. It also helped remind young people that the restrictions placed on their lives (e.g. parental supervision at mealtimes) was temporary – that they could get back to some of these things once some movement towards recovery was made.

This was especially difficult for young people to do in the early stages of treatment, when the illness could be all consuming and there was a strong ambivalence about letting others know that they might want to recover. Some examples of small steps that young people described as useful were things like being able to eat without supervision at school, recommencing sport, and reengaging with peers and social activities in a more independent fashion.

“I think what really helped me was to pinpoint specific things that I was missing out on and to have specific aims to look forward to”.

When I was allowed to, uh, go back to running … I realised I if I wanted to keep that up, I needed to, like, focus on food and stuff like that.

Nevertheless, this was not always possible. For some people, previously enjoyable activities were not motivating when the illness was at its strongest.

They always told me that I have to look out for motivators. But I mean, yes, I like something and I wish I was good so I could do it, but then I guess the desire of like wanting to stay the way I was, was a bit stronger than wanting to do sport ….

#### 4b. Making life bigger to squeeze out the illness

Even if life outside the illness did not feel motivating at the early stages, in the middle and later parts of treatment, typically once the young person had moved to a place of acceptance of recovery (cf. theme 3: through, not around – no way out), the importance of bringing life outside of the illness into treatment sessions more was described as important by nearly all participants. Moving away from increased parental support was described by many as a mixture of relief, excitement and worry – relief about increasing autonomy; anxiety that the illness would be too overwhelming to manage alone.

Like I was really scared at the beginning. But then it, like, actually was like it was much better than I thought it was gonna be, …I just felt like I had more kind of independence with it, and I kind of, felt that was the times where I learned, like, how to sort of deal with my anxiety and, like, stuff like that the best.

“Me having that agency, which initially was a bit intimidating. I was like, wow, I’m kind of in control when I’m eating, That’s, you know, that’s kind of a big step. But then, actually, I found that through that it really helps me”.

Over time giving me a bit more, when I had started to change my thoughts, giving me some space, some own freedom and independence to make more of my own choices and to make my own meals, I think definitely helped. Because one thing that was really emphasised in meetings was that practice and doing it over time, and then it will get easier. Which is definitely true, because at first it was difficult but doing it continuously every day really helped. So, I think that was a mixture of what we were told in the meetings and then that was implemented by my parents, so that really helped.

Getting back to life outside the illness was catalysed by different things for different people. For some, this was driven by the young person.

It probably took a little bit more time for them to trust me than it took for me to feel like I could trust myself around food.

For others, it was the therapist or family member encouraging independence.

Towards the middle to end of treatment, when we had the conversation with the therapist, she allowed me to be more independent, like make my own lunch to school and stuff like that. And that really helped, because I guess I kind of taught myself that I was able to do things on my own. Yeah, and gain that independence back.

Sometimes it was described as more circumstantial. For example, the ending of summer and the return to school, or working towards being able to go on a holiday.

I went to [country name] on holiday, and it was kind of scary because it was already pretty early on in treatment. And there was no way that I could have stuck to meal plan in there, it was complete buffet, like nothing you could track, nothing you could do this, that and the other. And I basically just had this mindset of just, why not? And I just kind of ate anything and everything. But I think that was a big turning point for me.

For others it was the social influence of being around others.

Although comparisons aren’t always a good thing, I think to an extent they are in a positive way because I could see how disordered my thoughts and ways were. So, it helped me to, I guess, realise that it’s just food. So, it helped me to realised, to gain some rationality, I guess.

Regardless of the catalyst, it was important that life outside the illness was actively brought into treatment by the MDT. It was important to pursue the life beyond, rather than waiting for it to happen.

#### 4c. Adjusting treatment to give space

Many also spoke of the importance of spreading out treatment sessions to allow for more space to reflect and practice managing independently without the safety net of treatment sessions. Increasing the amount of time between treatment sessions allowed for more engagement with life. This also acted as small practice runs of later discharge.

I think having the sessions spaced out more was really like, that I think helped the most, weirdly, because it meant I kind of had to deal with stuff on my own more than like having then relying on the sessions.

“During the end of treatment, the longer frequency between appointments really did help, because of course at first having them regularly was vital. But then because it was so frequent, sometimes it was always there, so always kind of coming back to it. So, it was very repetitive. So, I think just having some time away to kind of reflect and use things that they had suggested in meetings and be able to have a long period of time to figure that out on my own, really did help. And it also meant I could be more aware of all the things I was achieving within that time because I could think and reflect on it and then go back to the meeting afterwards and kind of tell them.”

One person described feeling like there were too many sessions and actually spacing them out more or having fewer might have helped to promote autonomy and recovery sooner. They said: “*I felt maybe I had a few too many appointments, where I kind of was just sat there, and I didn’t really have much to say.”*

#### 4d. Broadening the emotional world

Lastly, it was described as important to ensure that as soon as a more healthy routine of food and eating was established the treatment moved to the young person’s emotional and social world as much as possible. This helped to build trust and engagement in the process by signalling to the young person that treatment was going to take a holistic approach.

At the beginning, I didn’t really know exactly what I was feeling, partly because obviously when I needed to then gain the weight a bit … then you can think more clearly. So, then we talked a little bit later on more exactly about the emotions, when I could think exactly how I was feeling whereas we talked a bit more about the behaviours to begin with, but I think that was better in a way … that worked well for me.

For some this was quite shocking, but welcome. One person said: *“He [my therapist] asked me, like, what my values were, like life values. And I was really a bit taken aback.”* This surprise spoke to the importance of the therapist leading and persisting with this change if it is not led by the young person themselves.

## Discussion

The aim of this study was to explore young peoples’ perspectives on how change is perceived to occur during FT-AN. Four themes were generated that described different processes of change during treatment. All were inter-related and influenced each other. Three specific elements (an awakening; through, not around – no way out; the life beyond) were all described as combining to generate gradual initial behaviour change and later holistic change. With warm, persistent and consistent perseverance and support, the young people described being able to push through the distress of returning to more healthy eating patterns and physical health.

Trust in relationships with parents and clinicians in the MDT, as well as others in their social and educational circles, was described as the base from which these three elements were either enhanced or hindered. In a way, relationships acted as the foundation upon which the other elements were enacted. All young people in this study described how strained relationships could feel at the beginning of treatment. Yet, once a connection was established and trust was (re)built, individuals could start communicating more openly and experimenting with new behaviours together. For most, this process of learning and change resulted in a strengthening of relationships. At the beginning of treatment, nearly all young individuals faced a dilemma: while a part of them recognised a need for increased parental and family support, illness-related cognitions and anxiety about weight gain often led them to actively deny wanting it or reject that very support. Notably, warm, firm parental and team perseverance was said to be crucial to recovery. Even though the young people struggled to ask for support, parents’ warm, consistent perseverance sent a strong message of love and care.

These findings highlight that certain common preconceptions about FBT/FT-AN may not hold true for young people who have a relatively ‘good’ outcome. Specifically, current findings suggest a strong alliance with the therapist/MDT can be experienced by young people even early on in treatment and that this is predominantly marked by trust in the therapist/MDT, rather than voiced liking of them. Additionally, young people are eventually able to appreciate the methods implemented in the early phases as being in the service of their well-being and required for recovery. Relatedly, over time as the illness recedes and the young person’s identity becomes more of a focus in treatment, the young person may increasingly feel more valued and recognised in their entirety. They may also retrospectively understand that restrictions and high parental involvement were temporarily required for change to occur. While this might not hold true for those who do not have a good outcome (cf. Conti et al. 2021; Medway and Rhodes 2016), if FT-AN is implemented in a person-centred, formulation driven manner the concerns about treatment not feeling holistic enough may be avoided.

The current study suggests these four key themes are important to target in treatment directly and explicitly. They map relatively closely onto the theoretical model underpinning FT-AN (Blessitt et al. 2020; Eisler et al. 2016). In the FT-AN model containing and validating family anxiety is crucial. By taking a position of expertise in eating disorders, the MDT promotes confidence in the team and establishes a secure base for treatment. Parental firmness, especially around feeding, should go hand in hand with warmth and validation. It should be seen as care, not control (Eisler et al. 2016). Baudinet, Simic, et al. (2021b) emphasised these points in their recent description of how the FT-AN model has changed over time.

There is also an emphasis in the FT-AN model on the importance of the therapeutic relationship changing during treatment; moving from a place of expertise in phase 1 and 2 to a more exploratory relationship in phase 3 and 4. During the latter phases, the therapist intentionally begins to help the young person and family manage uncertainty to support the young person in reclaiming developmentally appropriate responsibility and agency (Blessitt et al. 2020; Eisler et al. 2016). During the later phases, the therapist intentionally begins to help the young person and family manage uncertainty.

What the current findings do emphasise is that including the young person and doing everything possible to gain their trust is important and should not be too quickly abandoned or equated with freely permitting the young person to engage in unhealthy illness behaviours. Rather, trust seems to be built through warmth, knowledge and consistent perseverance - a sense that the family and team care too much to let illness behaviours dominate, rather than the young person being controlled unnecessarily. This is a subtle but important distinction in supporting the families to work together in FT-AN. The current data also emphasises how crucial it is for clinicians and parents/caregivers to persevere with engagement efforts, even in the face of voiced resistance and distress. This is important to emphasise given recent descriptions of FBT that describe the young person as ‘not actively involved’ in treatment (Dalle Grave et al. 2019).

There were a few young people in the study who described making behaviour change (e.g. weight gain) but still continued to struggle with the psychological symptoms of the illness. For these young people, there was a sense that they did not or could not engage as much in the emotional aspects of treatment. This further pointed to the importance of engagement from the young person in treatment in order to make a more holistic recovery.

The emphasis on engagement and trusting relationships in the current data mirrors how young people and emerging adults (age 15–26 years) described their experience of compulsory treatment for anorexia nervosa in an earlier study. Tan et al. (2010) reported that the perception of involuntary treatment for AN was moderated by the level of perceived respect and trust in relationships with family members and the clinical team. Essentially, *what* happened in treatment could be perceived as helpful or unhelpful, respectful or disrespectful, depending on the *manner* it was delivered in.

The current findings also fit with qualitative data exploring the perceived change mechanisms within more intensive, FT-AN-informed treatments, such as multi-family therapy (cf. Baudinet et al. 2021c for review) and day programme treatment (cf. Baudinet and Simic 2021 for review). These interventions are often provided when things ‘feel stuck’ in FT-AN. In both multi-family therapy and intensive day programme treatment the importance of trust, containment and increased support are seen as important in enhancing treatment response and helping families break maintenance factors (Baudinet et al. 2023a; Baudinet et al., in press; Colla et al. 2023; Gledhill et al., 2023). Perhaps one marker of the need for intensive treatment is when one or more of the generated themes in the current study are not able to be activated in standard outpatient FT-AN. This is a question for future research.

### Strengths and limitations

One strength of this study is the inclusion of the young peoples’ voices alone. Rarely is this reported in the literature (often studies report on parent or combined parent and child experiences). This gives a better understanding of the experience of the person going through treatment. Additionally, not all young people in the study described making a full psychological recovery, which gave insights into potential differences in treatment mechanisms and what might be missing for these young people.

There are also several limitations to the current study. The sample size, although appropriate for a qualitative study, is still relatively small and only describes the experience of a small, relatively non-diverse group of people. Most were cisgendered White females, all of whom returned to a prelatively hysically healthy state and all of whom received FT-AN at the same treatment centre. It will be important to understand whether these experiences are replicated by those in other services and treatment contexts and with a more varied treatment response. All young people in this study gained weight and most described psychological improvements. As such these data represent a cohort of people for whom treatment was relatively effective.

Additionally, only approximately one third of eligible young people who were approached participated in the study and very little family/parent demographic data are available. This might suggest a selection bias. While no differences in some baseline and treatment factors were reported, it is possible other differences existed between these groups that were not reported, which influenced the current findings. Understanding what happens for more ethnically diverse young people, those who do not weight restore and/or those who do not complete treatment is important to address in future research. Furthermore, not only was this study based on patient perceptions, but on retrospectively gathered data. It could be interesting for future qualitative research to conduct a series of prospective briefer interviews at key junctures of treatment. To minimise bias, all transcripts were de-identified during the transcription process. Lastly, one of the analysing authors (JB) was also involved in treatment delivery for some of the young people and the other analysing author (AK) was not a practitioner of FT-AN. These experiences inextricably influence the findings.

## Conclusion

The current study aimed to explore the process of change during FT-AN from the perspective of young people who have received treatment. Themes generated from the data suggest several factors are key to promoting change in FT-AN; (1) emphasising engagement with all family members from the outset, (2) ensuring life outside the eating disorder is always brought into treatment sessions, (3) supporting the young person to understand their illness form different perspective and recognise its impact, (4) creating a home and treatment environment in which the illness cannot be avoided and will be addressed. Empirical investigation of these elements is warranted.

## Data Availability

Data will be made available upon reasonable request for those participants who have consented to this.

## Abbreviations

%mBMI: Percentage of median body mass index
FBT: Family-based treatment
FT-AN: Family therapy for anorexia nervosa
NICE: National Institute for Health and Care Excellence, UK
PFT: Parent focused therapy
